# Supplementary material for: ProteinShader: illustrative rendering of macromolecules
Source: BMC Struct Biol. 2009 Mar 30;9:19. doi: 10.1186/1472-6807-9-19 (PMC2672931; doi:10.1186/1472-6807-9-19)
Supplement: Additional file 1 — ProteinShader program without source code. This compressed file contains the complete ProteinShader program including associated libraries, but no source code. A README.txt file gives an overview of the ProteinShader distribution, and the index.html file in the help subdirectory has directions on getting started with the program as well as a set of tutorials. [file 1472-6807-9-19-S1.zip › ProteinShader-beta-0_9_4-binary/help/tutorials/1BL8.html]

ProteinShader Tutorial: 1BL8


# ProteinShader Tutorial


♦ Home


♦ Download


♦ Getting Started

Choose a Link:

Installation

Running

Troubleshooting

PDB Files


♦ Tutorials

Choose a Link:

List of Proteins

Style Options

Saving Images


♦ General Controls

Choose a Link:

Menu Bar

Keyboard Shortcuts

Mouse Motion

Motion Panel

Antialiasing


♦ Cartoon Controls

Choose a Link:

Control Panels

Selection Panel

Radio Buttons

Decorations Panel

Adding Textures

Color Panel

Visibility Panel

Side Chains Panel


♦ Atom Controls

Choose a Link:

Control Panels

Selection Panel

Radio Buttons

Color Panel

Visibility Panel

Scale Panel

Tiling Panel


♦ Developers

Choose a Link:

Compiling

Running

Code Docs

Developer Links


## Potassium Channel: Transmembrane Alpha-Helices [PDB:1BL8]

The potassium channel is an integral membrane protein composed of four subunits
that form a single central pore that is selectively permeable to K+ ions
[1, 2]. Each subunit is
a single polypeptide chain that includes three alpha-helices: two transmembrane
helices and one that faces the pore. The *Streptomyces lividans*
potassium channel, which has sequence similarity to all known K+ channels,
has been solved using x-ray crystallography
[3, 4],
and can be loaded loaded into the ProteinShader program by selecting Open from
the File menu
and then using the file chooser box to select the 1BL8.pdb file.


## Cartoon-style displays with halftoning

When the potassium channel protein is first loaded, it will be displayed as a
pen-and-ink style drawing of ribbons and tubes. To obtain something closer to
the images in Figure 1, first go to the
Style menu
above the canvas and select Ribbons from the Cartoon submenu. Then go to the
Decorations subpanel,
and with Model showing in the menu under the Selected radio button,
select Vertical Bars from the Halftone Texture menu and None from the
Bend Texture menu. To fade out the loop regions, switch from the
Decorations subpanel
to the
Cartoon Visibility subpanel
and click on the Loops radio button before pressing the Translucent button.
The degree of translucency can be adjusted with the slider control below
the button.

The structure of the potassium channel is roughly cone-shaped, and has been
compared to the petals of a flower that opens towards the outside of the cell
[1],
as shown in Figure 1A. To obtain this same view, first go to the
Orientation menu
above the canvas and select Back, and then left-click with the mouse
and drag horizonally across the canvas to the left for a short distance. The
center mouse button (or scroll wheel) can be used to zoom in a little, and
the right mouse button can be used to translate the entire image up or down
or side to side (see the
Mouse Motion Control section
for details and for Macintosh single-button mouse options).

The crystal structure of the potassium channel has three K+ ions and one water
in the channel. To see these atoms, first go to the
Visibility menu
above the canvas and select Heterogens Allowed and then Waters Allowed. In the
orientation shown in Figure 1B, only one of the K+ ions can be seen, so use the
Orientation menu
above the canvas to select Left, and then drag the mouse vertically downwards
on the canvas a short distance to get something similar to Figure 1C, where
all three K+ ions plus the oxygen of a water molecule are visible.


|  |  |  |
| --- | --- | --- |
| **A** | **B** | **C** |

**Figure 1.** Ribbons style display of the potassium channel, a
transmembrane protein with an overall shape roughly like a cone.
(A) The larger diameter end of the cone as seen from outside of the cell.
Alpha-helices are shown as wide ribbons, while loop regions are translucent.
(B) Same as part A, but with a K+ ion visible in the channel.
(C) Same as part B, but with a side view where the end of the channel that
faces outside the cell is on the right. Three K+ ions (deep pink) and a single
water (red) inside the channel are now visible (hydrogen atoms are not shown).
The images were captured as described in the
Saving Images
section of the tutorials main page.


## References

1.
Brandon C, Tooze J: **Membrane Proteins.**
In *Introduction to Protein Structure.* 2nd edition.
New York: Garland Publishing; 1998: 223-250.

2.
MacKinnon R:
**Potassium channels.**
*FEBS Lett* 2003, **555**: 62-65.

3.
Doyle DA, Morais Cabral J, Pfuetzner RA, Kuo A, Gulbis JM, Cohen SL,
Chait BT, MacKinnon R:
**The structure of the potassium channel: molecular basis of
K+ conduction and selectivity.**
*Science* 1998, **280**: 69-77.

4.
**Potassium channel PDB entry 1BL8**
[http://www.rcsb.org/pdb/explore/explore.do?structureId=1BL8]


---

|  |  |  |  |
| --- | --- | --- | --- |
| Hosted by |  |  |  |

Webmaster: Joe Weber,
joe.weber AT alumni.duke.edu
  
Web site last updated: December 30th, 2008
  
© 2007–2008 Joseph R. Weber
